# Supplementary material for: Neoadjuvant therapy with immune checkpoint blockade, antiangiogenesis, and chemotherapy for locally advanced gastric cancer
Source: Nat Commun. 2023 Jan 3;14:8. doi: 10.1038/s41467-022-35431-x (PMC9810618; doi:10.1038/s41467-022-35431-x)
Supplement: Supplementary file 1 — Supplementary Information [file 41467_2022_35431_MOESM1_ESM.pdf]

Supplementary information

Supplementary Figures 1-16

Supplementary Figure 1. CONSORT diagram.

Supplementary Figure 2. Multiplex immunofluorescence staining of patients P10 and P20 who received non-MPR.

Supplementary Figure 3. Adverse events of patients undergoing neoadjuvant treatment.

Supplementary Figure 4. Postoperative complications of patients.

Supplementary Figure 5. Genomic changes during neoadjuvant therapy.

Supplementary Figure 6. VAF values of nonsynonymous mutations in pre- and post-treatment samples.

Supplementary Figure 7. Dynamic changes in cellular frequencies (CCF) of nonsynonymous mutations from pre- to post-neoadjuvant treatment.

Supplementary Figure 8. Differentially expressed genes and functional enrichments.

Supplementary Figure 9. Baseline levels of immune-related genes between PPR+ and non-PPR+ groups.

Supplementary Figure 10. Changes in immune-related gene expression.

Supplementary Figure 11. Baseline levels of immune-related signatures between PPR+ and non-PPR+ groups.

Supplementary Figure 12. Changes in immune-related signatures.

Supplementary Figure 13. Changes in proportions of immune-related cells in PPR+ (A) and non-PPR+ patients (B), estimated by xCell.

Supplementary Figure 14. Immunophenotypes of paired specimens.

Supplementary Figure 15. TCR repertoire overlaps across samples.

Supplementary Figure 16. Dynamic changes in D50 (A), Shannon (B), and evenness (C) index from pre- to post-treatment samples.

Supplementary Tables 1-3

Supplementary Table 1. Baseline characteristics of patients and treatment medicines.

Supplementary Table 2. TCR characteristics.

Supplementary Table 3. Predictive values of putative biomarkers.

SUPPLEMENTARY FIGURES

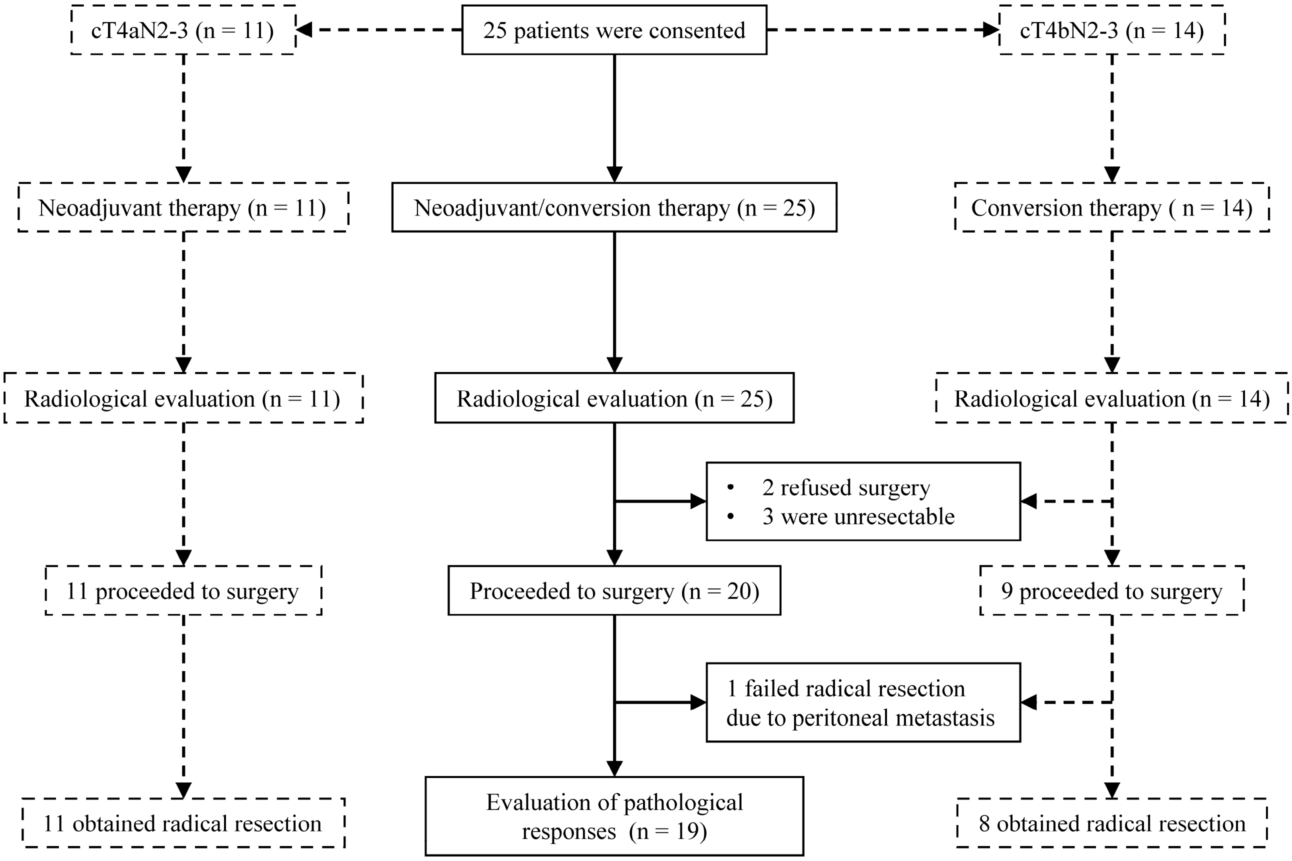

Supplementary Figure 1. CONSORT diagram. The diagram shows the progress through this trial. Solid lines indicate all recruited patients, and dotted lines indicate cT4a and cT4b patients separately.

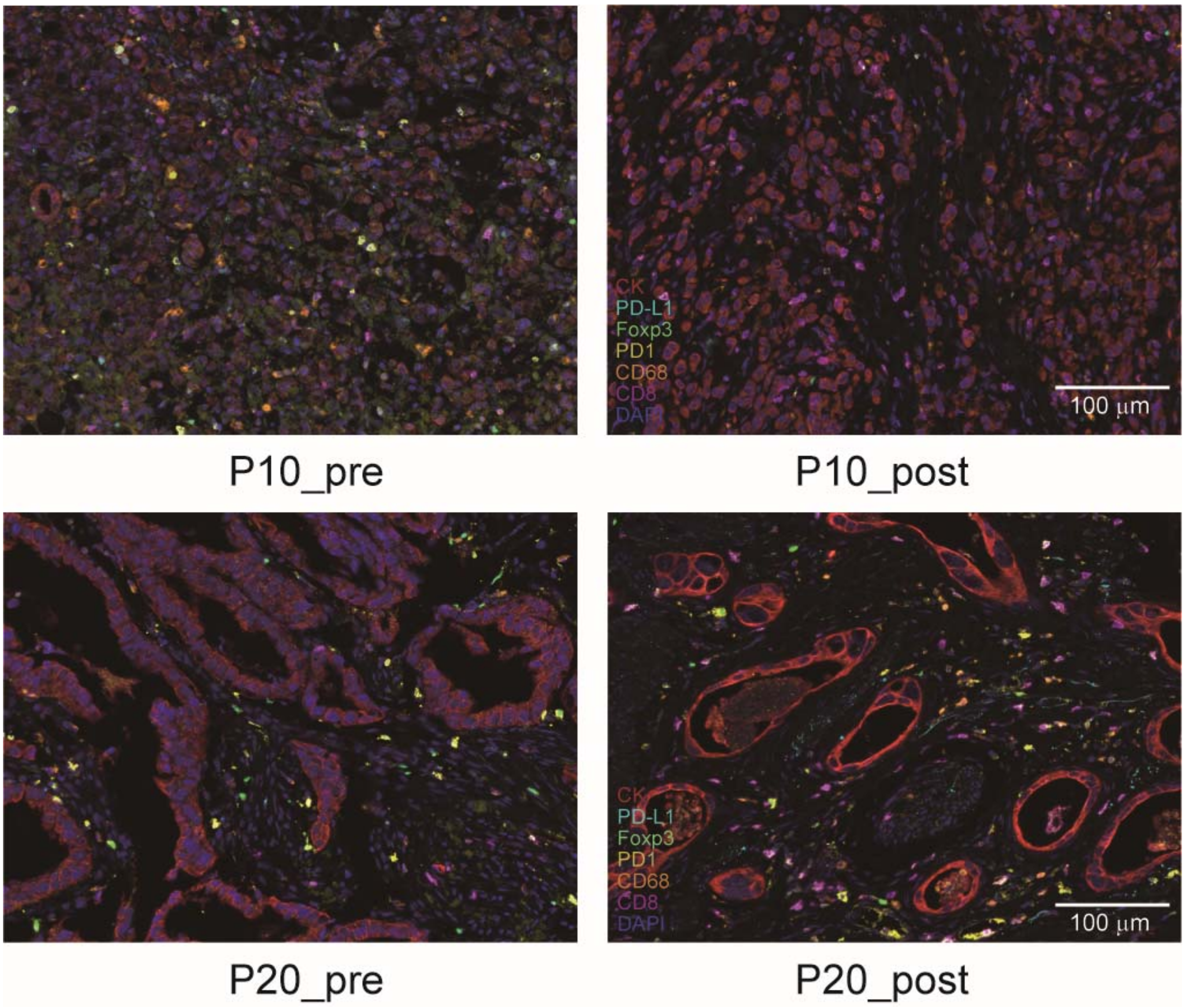

Supplementary Figure 2. Multiplex immunofluorescence staining of patients P10 and P20 who received non-MPR. Visible structures include cytokeratin-positive tumor cells (red), PD-L1+ cells (cyan), FoxP3+ regulatory T cells (green), PD-1+ cells (yellow), CD68+ macrophages (orange), CD8+ T cells (magenta), and nuclei (blue).

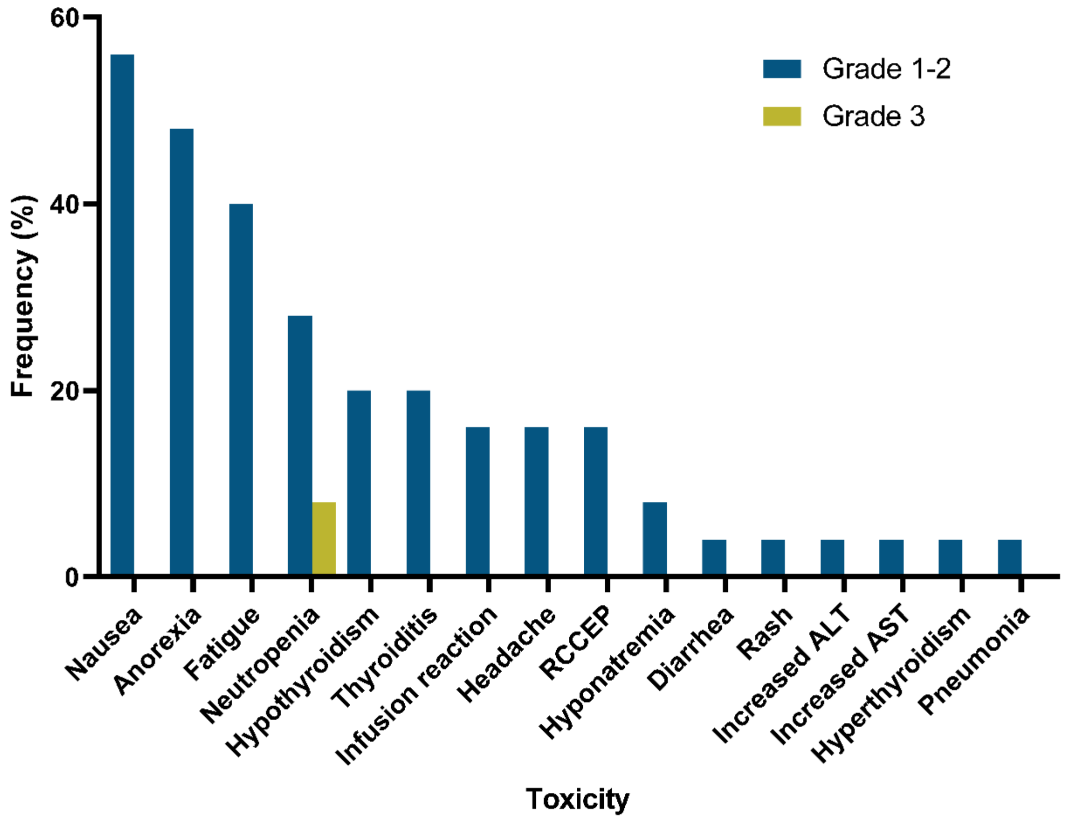

Supplementary Figure 3. Adverse events of patients undergoing neoadjuvant treatment (n = 25).

No grade 4-5 toxicity occurred. RCCEP, reactive cutaneous capillary endothelial proliferation.

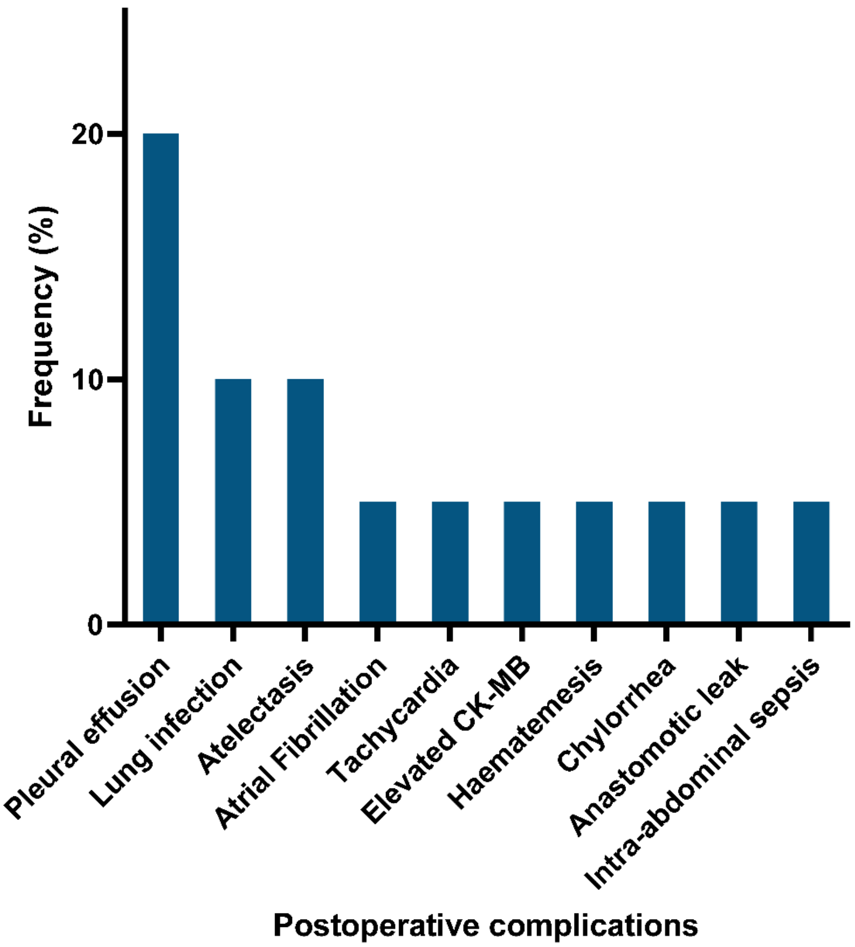

Supplementary Figure 4. Postoperative complications of patients (n = 20). All complications were grade 1-2, and no grade 3-5 complications occurred, according to the Clavien-Dindo classification.

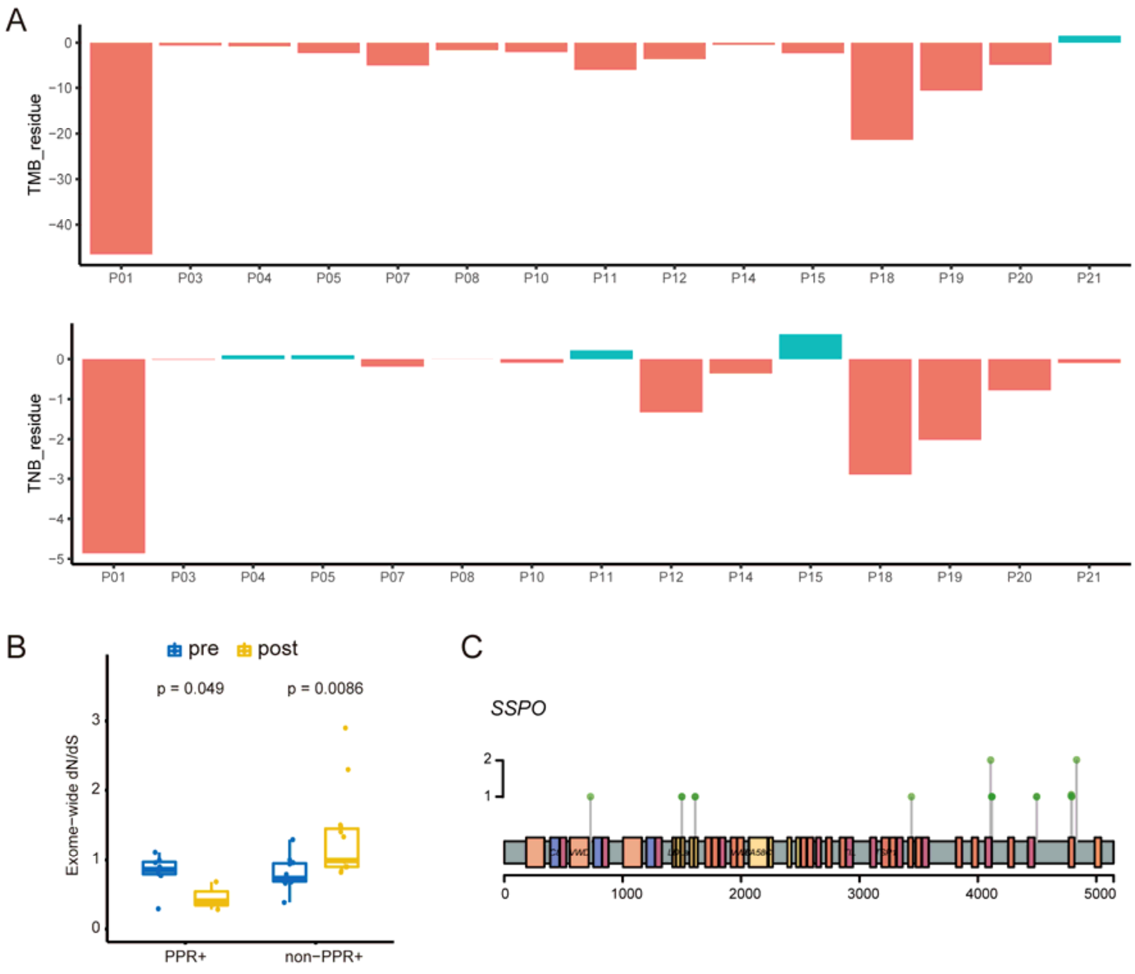

Supplementary Figure 5. Genomic changes during neoadjuvant therapy. A Changes in TMB and TNB indices after treatment in all patients (n = 15). **B** Differences in relative rates of nonsynonymous and synonymous mutations (dN/dS) between pre- and post-treatment samples in PPR+ (n = 8 and 3) and non-PPR+ (n = 11 and 11) patients. Wilcoxon rank-sum test was used for comparison. **C** Distribution of mutations in the SSPO gene.

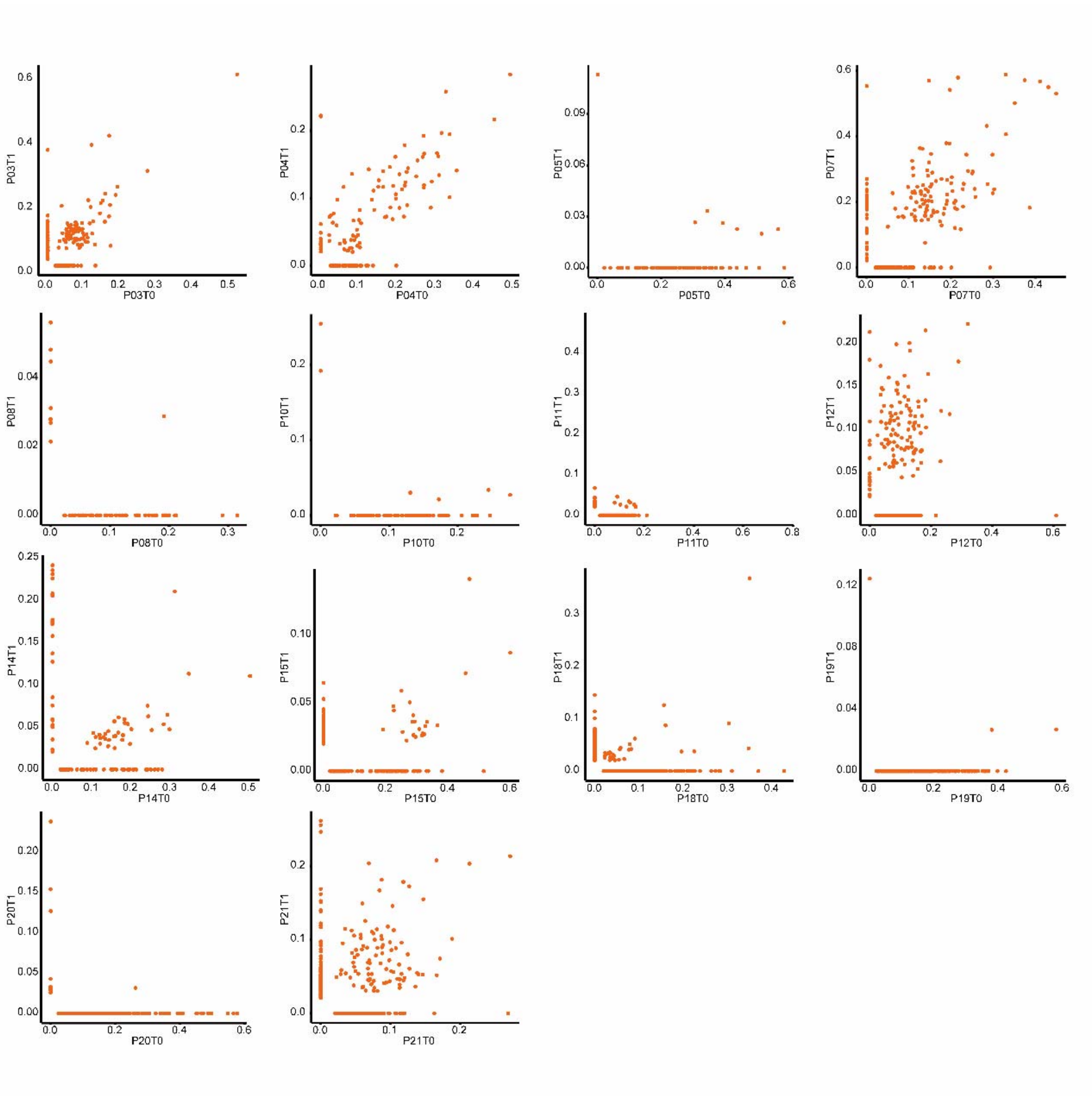

Supplementary Figure 6. VAF values of nonsynonymous mutations in pre- and post-treatment samples (n = 14).

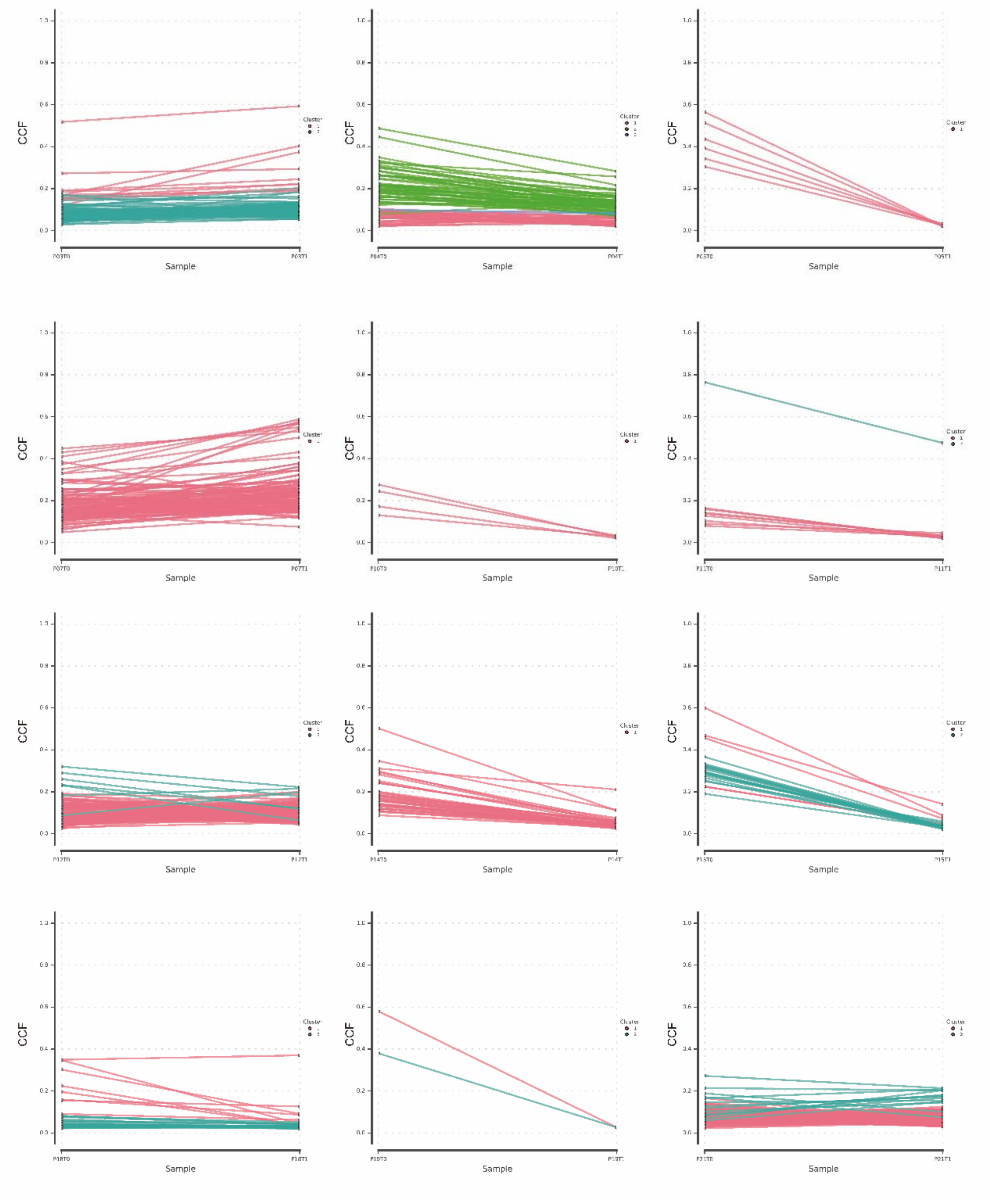

Supplementary Figure 7. Dynamic changes in cellular frequencies (CCF) of nonsynonymous mutations from pre- to post-neoadjuvant treatment (n = 12).

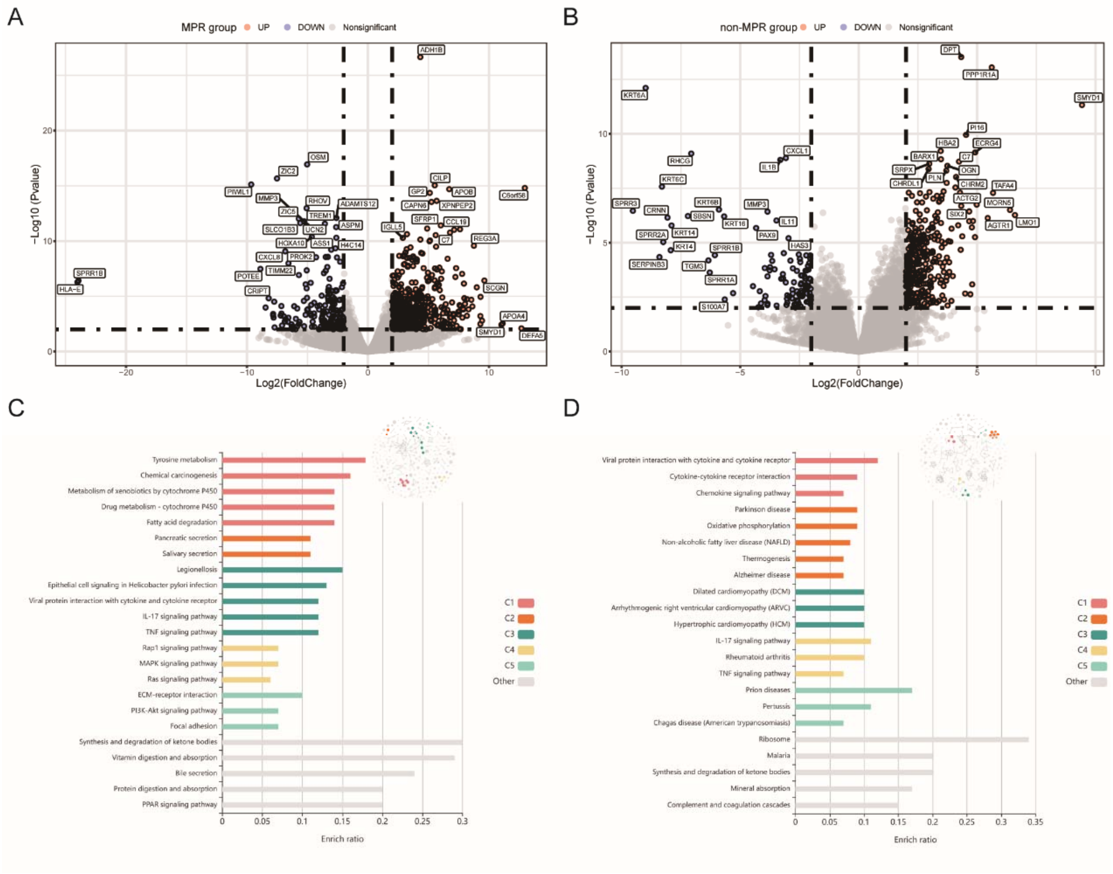

Supplementary Figure 8. Differentially expressed genes and functional enrichments. A Volcano plot showing the differentially expressed genes between pre- (n = 2) and post-treatment (n = 2) samples in the MPR group. **B** Volcano plot showing the differentially expressed genes between pre- (n = 10) and post-treatment (n = 12) in the non-MPR group. Color dots denote genes passing the P-value and fold change thresholds in A-B. **C** Functional pathway enrichment by differentially expressed genes in the MPR group. **D** Functional pathway enrichment by differentially expressed genes in the non-MPR group. Similar pathways are clustered and shown in different colors in **C-D**.

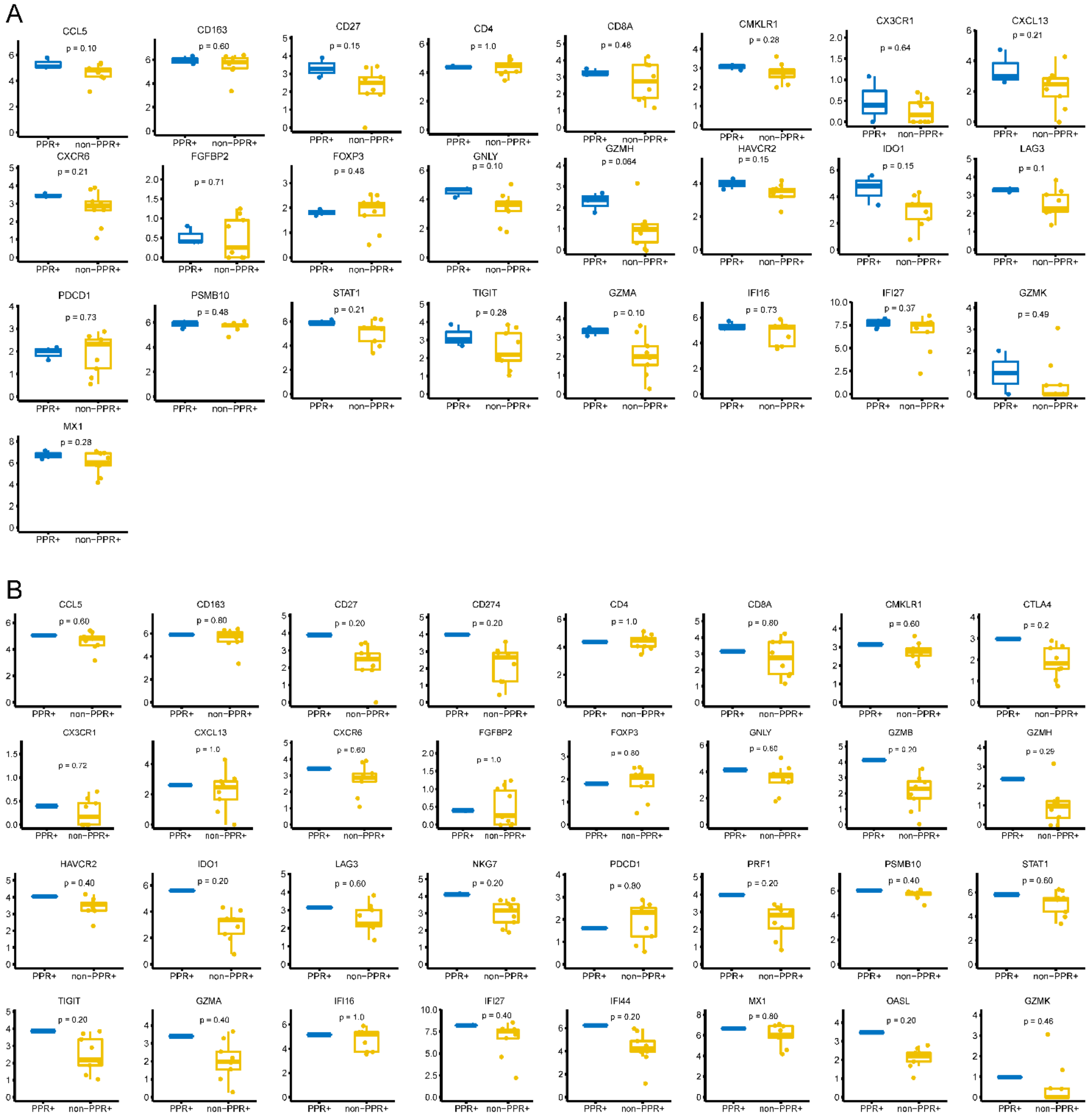

Supplementary Figure 9. Baseline levels of immune-related genes between PPR+ and non-PPR+ groups. A. Differences in baseline immune-related gene levels between PPR+ (n = 3) and non-PPR+ (n = 9) groups. **B.** Differences in baseline immune-related gene levels between PPR+ (n = 1) and non-PPR+ (n = 9) groups in MSI-H-excluded population. Boxplots represent median, 25th, and 75th percentiles. Wilcoxon rank-sum test was used for comparison in **A** and **B**.

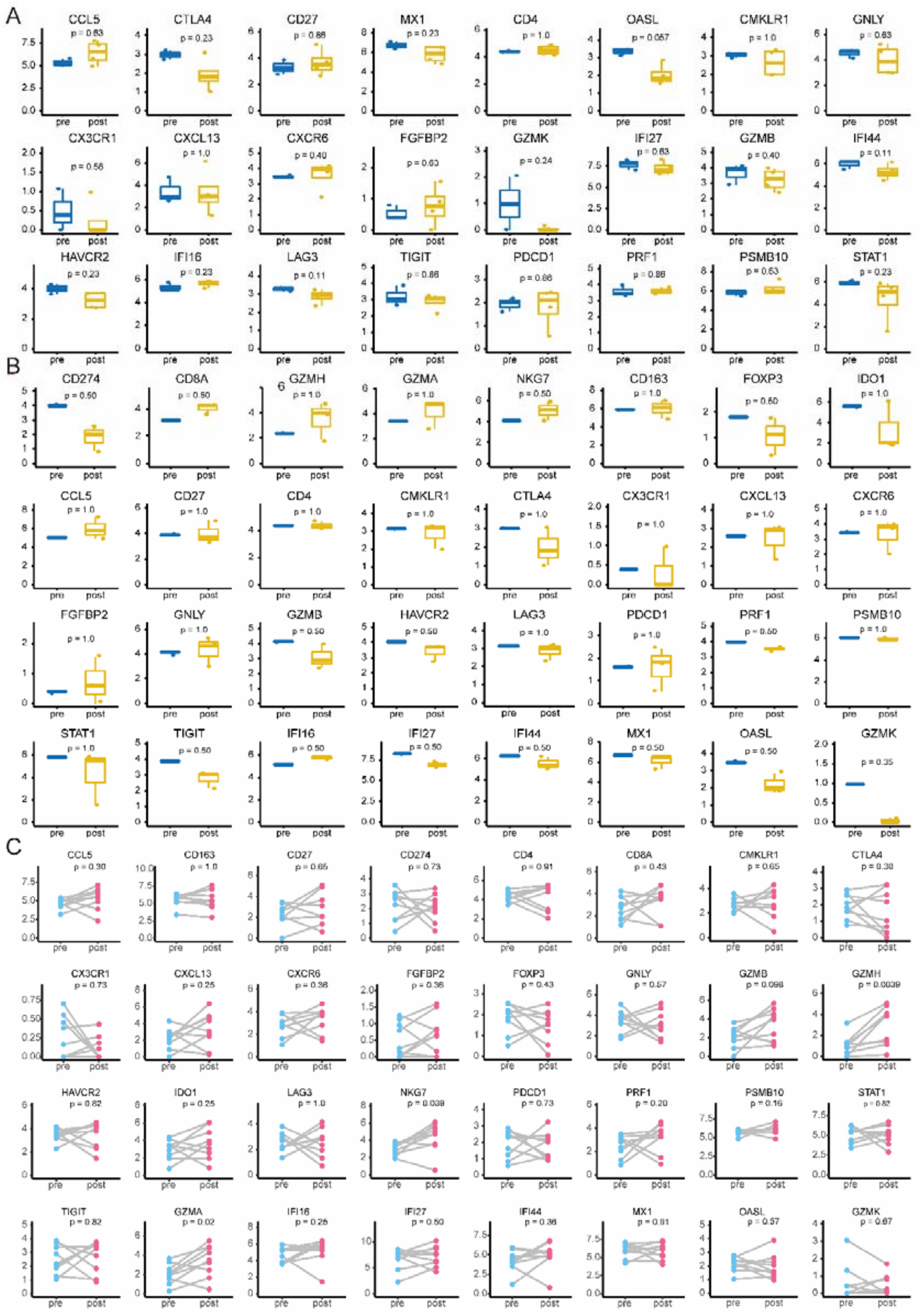

Supplementary Figure 10. Changes in immune-related gene expression. **A** Differences in immune-related gene levels between pre- (n = 3) and post-treatment (n = 4) samples in the PPR+ group. **B** Differences in immune-related gene levels between pre- (n = 1) and post-treatment (n = 3) samples in the PPR+ group with MSS. Wilcoxon rank-sum test was used for comparison in **A** and **B**. C Differences in immune-related gene levels between pre- and post-treatment samples in the non-PPR+ group (n = 9). Wilcoxon signed-rank test was used. Boxplots represent median, 25th, and 75th percentiles.

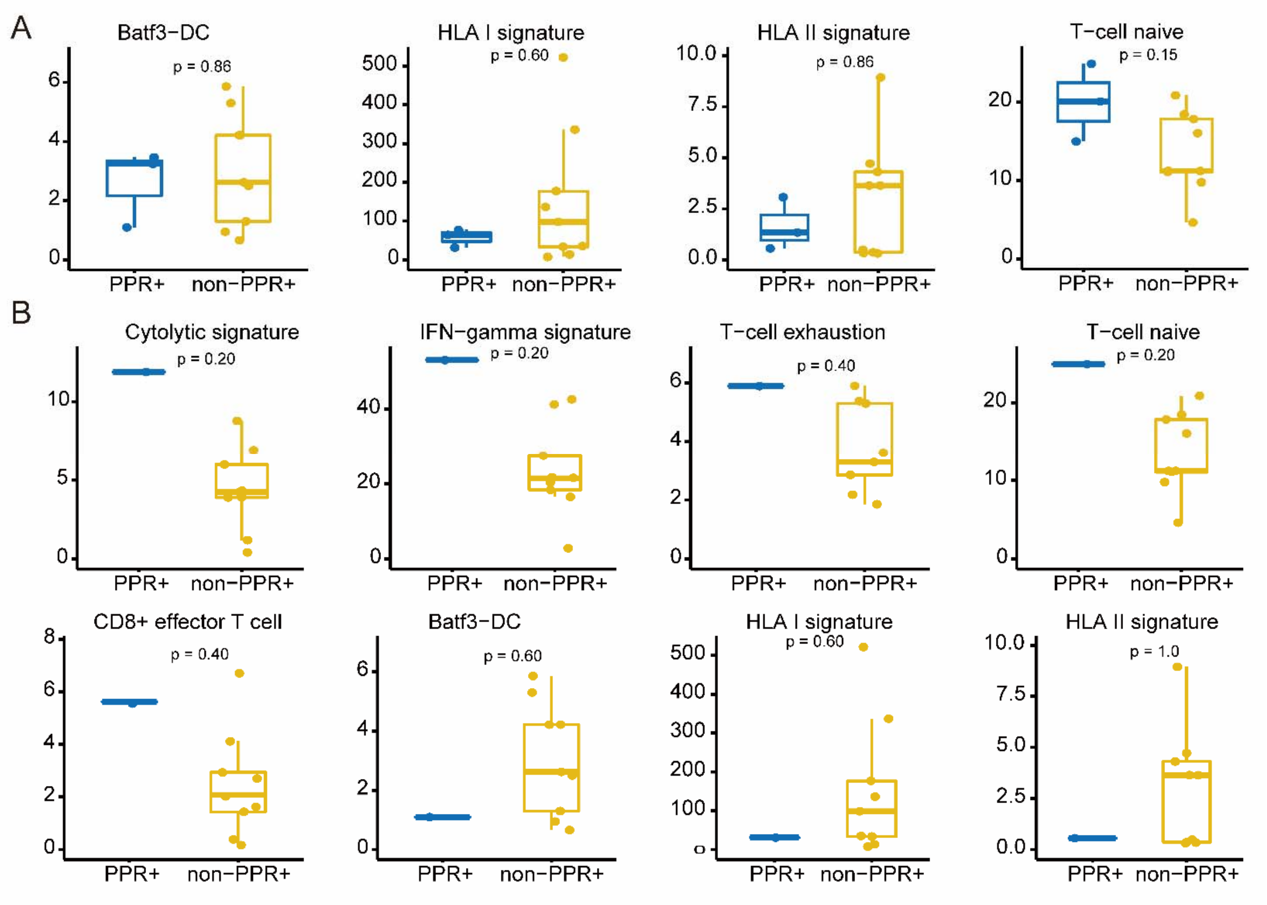

Supplementary Figure 11. Baseline levels of immune-related signatures between PPR+ and non-PPR+ groups. A. Differences in baseline immune-related signatures between PPR+ (n = 3) and non-PPR+ (n = 9) groups. **B.** Differences in baseline immune-related signatures between PPR+ (n = 1) and non-PPR+ (n = 9) groups in MSI-H-excluded population. Wilcoxon rank-sum test was used for comparison in **A** and **B**.

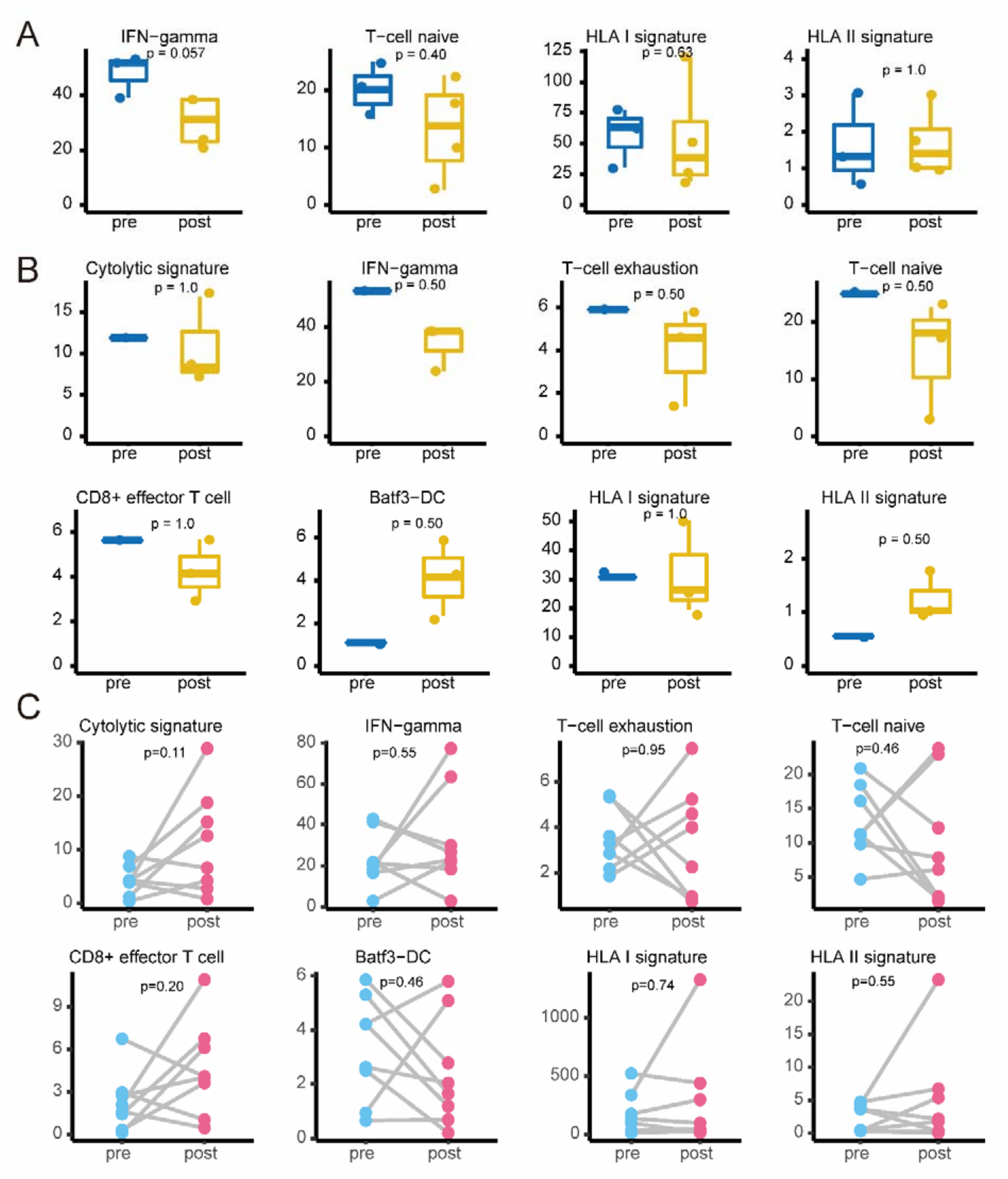

Supplementary Figure 12. Changes in immune-related signatures. A Differences in immune-related signatures between pre- (n = 3) and post-treatment samples in PPR+ (n = 4) patients. **B** Differences in immune-related signatures between pre- (n = 1) and post-treatment (n = 3) samples in PPR+ patients with MSS. Wilcoxon rank-sum test was used for comparison in **A** and **B**. Boxes represent median, 25th, and 75th percentiles in **A** and **B**. **C** Differences in immune-related signatures between pre- and post-treatment samples in non-PPR+ patients (n = 9). Wilcoxon signed-rank test was used.

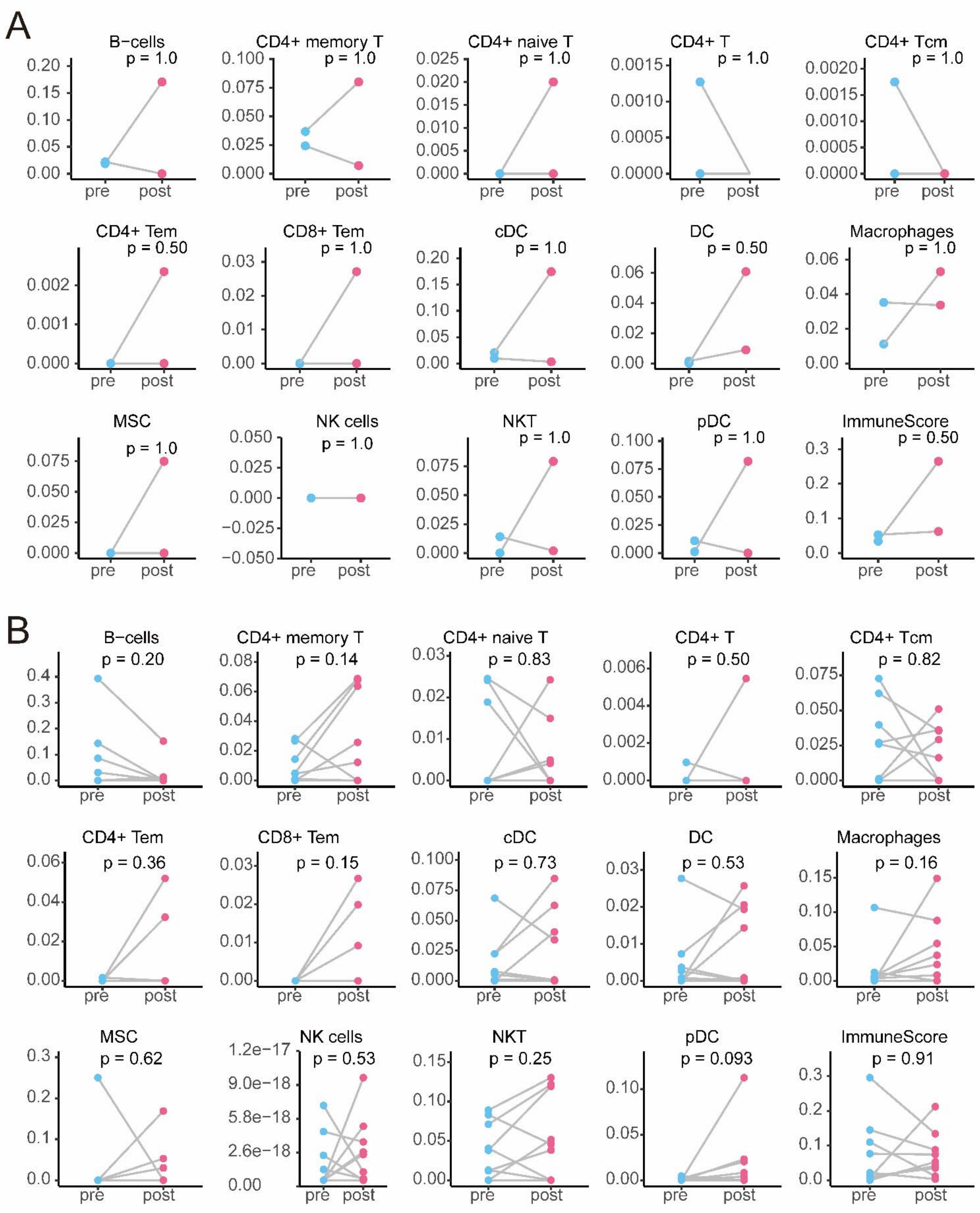

Supplementary Figure 13. Changes in proportions of immune-related cells in PPR+ (A, n = 2) and non-PPR+ patients (B, n = 9), estimated by xCell. Wilcoxon signed-rank test was used for comparison in A and B.

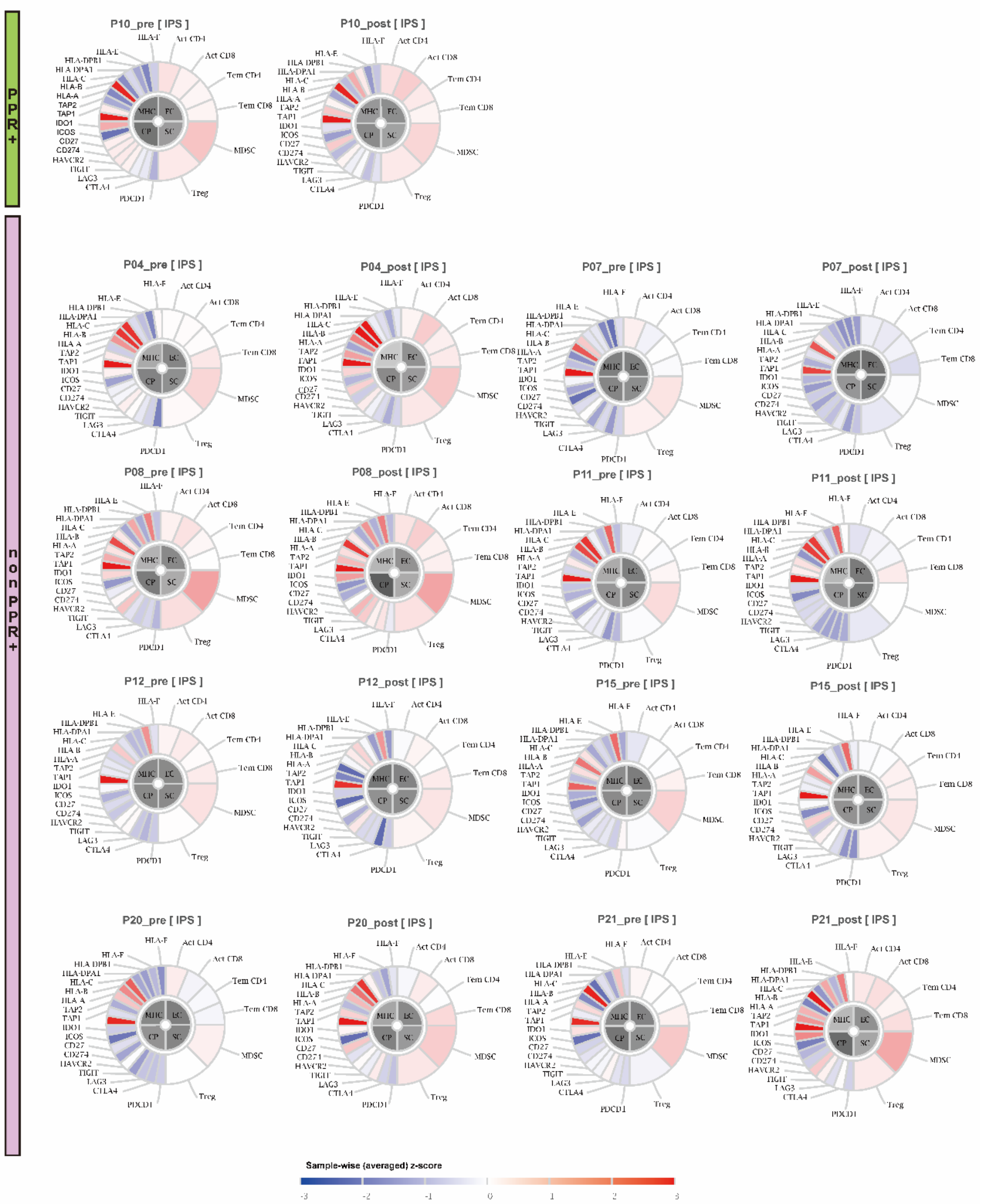

Supplementary Figure 14. Immunophenotypes of paired specimens. The major determinants are involved in four categories: MHC molecules (MHC), immunomodulators (CP), effector cells (EC), and suppressor cells (SC). n= 9.

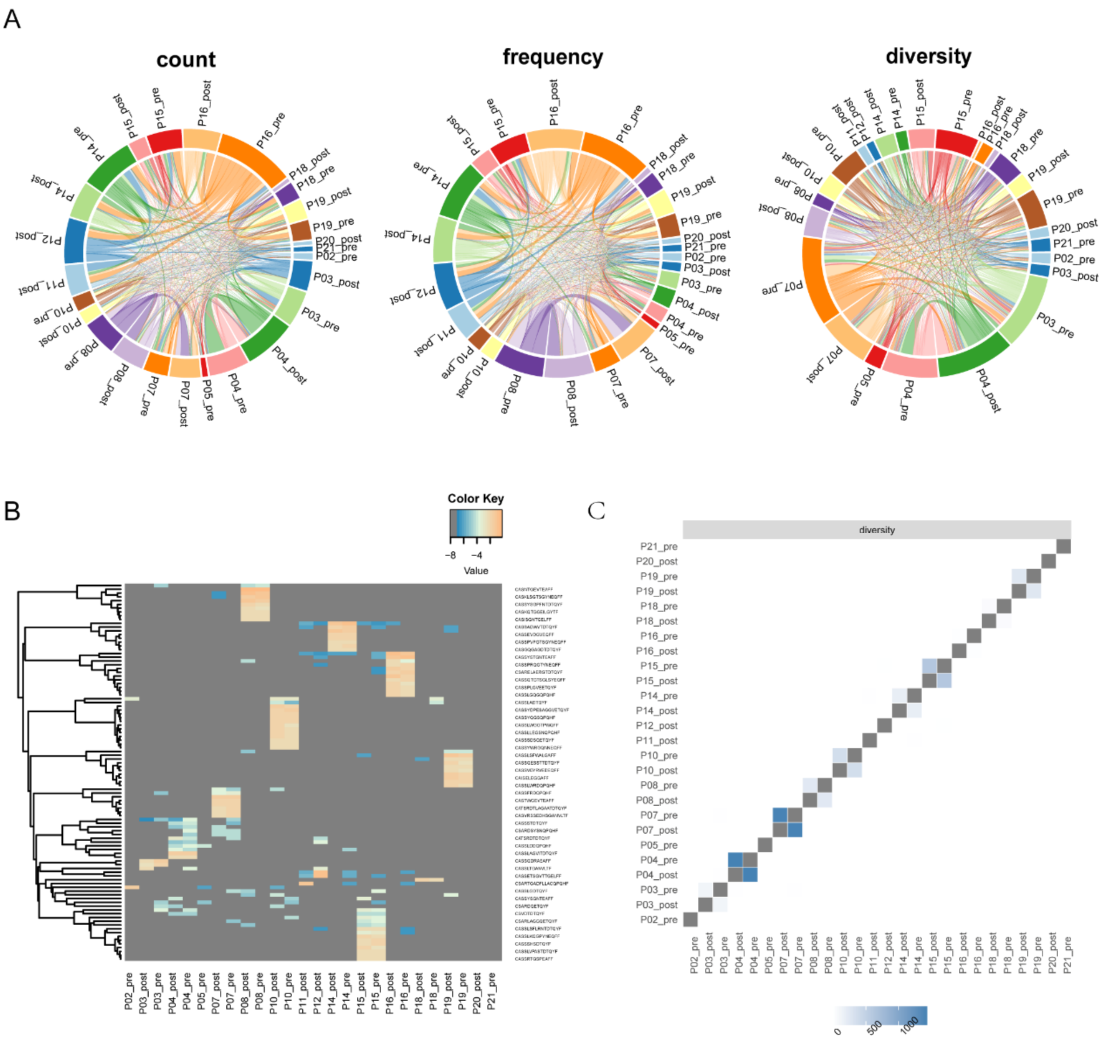

Supplementary Figure 15. TCR repertoire overlaps across samples in 16 patients. A Pairwise overlap circos plots showing counts (left) and frequencies (right) that are shared between samples. **B** Heatmap illustrating abundance of the top TCR clonotypes. **C** Heatmap illustrating correlation with diversity across samples.

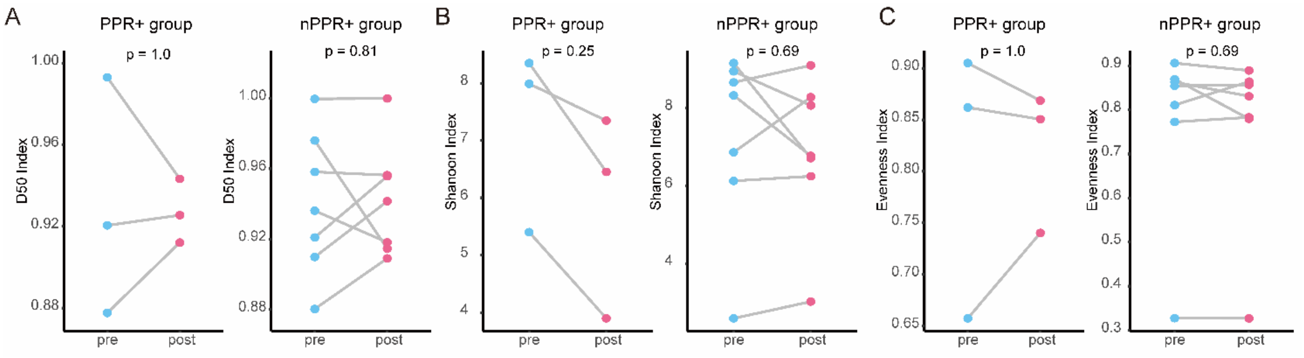

Supplementary Figure 16. Dynamic changes in D50 (A), Shannon (B), and evenness (C) indices from pre- to post-treatment samples. Boxplots represent median, 25th, and 75th percentiles. Wilcoxon signed-rank test was used.

SUPPLEMENTARY TABLES

Supplementary Table 1. Baseline characteristics of patients and treatment medicines.

Patient	ECOG	T stage	MSI	PD-L1 CPS	Treatment*	Cycles
P01	1	cT4a	MSI-H	0	cam+apa+S-1	2
P02	1	cT4a	MSI-H	60	cam+apa+S-1	2
P03	0	cT4a	MSS	0	cam+apa+S-1	2
P04	0	cT4b	MSS	0	cam+apa+S-1	2
P05	0	cT4b	MSS	NA	cam+apa+S-1	2
P06	1	cT4b	MSS	0	cam+apa+S-1	2
P07	0	cT4b	MSS	0	cam+apa+S-1	2
P08	0	cT4a	MSS	0	cam+apa+S-1	2
P09	1	cT4b	MSS	0	cam+apa+S-1	4
P10	0	cT4a	MSS	0	cam+apa+S-1	2
P11	0	cT4a	MSS	0	cam+apa+S-1	2
P12	1	cT4a	MSS	0	cam+apa+S-1	2
P13	0	cT4b	MSS	0	cam+apa+S-1	2
P14	0	cT4a	MSS	0	cam+apa+S-1+oxa	2
P15	0	cT4a	MSS	0	cam+apa+S-1+oxa	2
P16	0	cT4a	MSS	2	cam+apa+S-1+oxa	2
P17	1	cT4b	MSS	0	cam+apa+S-1+oxa	4
P18	1	cT4a	MSS	0	cam+apa+S-1+oxa	2
P19	0	cT4b	MSS	0	cam+apa+S-1+oxa	2
P20	1	cT4b	MSS	0	cam+apa+S-1+oxa	6
P21	1	cT4b	MSS	0	cam+apa+S-1+oxa	2
P22	0	cT4b	MSI-H	5	cam+apa+S-1+oxa	2
P23	0	cT4b	MSI-H	0	cam+apa+S-1+oxa	2
P24	1	cT4b	MSS	0	cam+apa+S-1+oxa	4
P25	1	cT4b	MSS	0	cam+apa+S-1+oxa	6

ECOG = Eastern Cooperative Oncology Group, MSI = microsatellite instability, MSI-H = MSI high, MSS = MSI stable, cam = camrelizumab, apa = apatinib, oxa = oxaliplatin.

* Treatment doses: camrelizumab: 200 mg d1; apatinib: 250 mg qd d1-14; S-1: 50 mg bid d1-10; oxaliplatin: 85 mg/m^2 d1.

Supplementary Table 2. TCR characteristics.

Sample Id	Count	Diversity	Mean cdr3nt length	Mean insert size	Mean ndn size	Convergence	d50 Index	Shanoon index	Evenness
P02_pre	3806000	4286	43.56	4.55	12.34	1.03	0.92	7.23	0.86
P03_post	20416252	3231	42.97	4.16	11.85	1.03	0.94	6.71	0.83
P03_pre	21876383	40589	42.62	4.09	11.6	1.04	0.91	9.16	0.86
P04_post	29504724	27786	43.19	4.4	12.1	1.05	0.91	9.1	0.89
P04_pre	13192764	14154	43.32	4.43	12.13	1.04	0.88	8.66	0.91
P05_pre	3876491	6611	43.9	4.7	12.88	1.05	0.93	7.51	0.85
P07_post	4285650	12290	43.26	4.27	12.07	1.05	0.91	8.07	0.86
P07_pre	2935907	35249	43.52	4.35	12.2	1.05	0.98	8.94	0.85
P08_post	2677615	10217	41.33	2.06	13.41	1.05	1	3.02	0.33
P08_pre	4203001	2732	41.23	1.75	13.54	1.04	1	2.59	0.33
P10_post	3721399	5642	43.53	4.38	12.44	1.04	0.93	7.35	0.85
P10_pre	5251434	10593	43.56	4.7	12.7	1.03	0.92	7.99	0.86
P11_post	5017370	2908	44.91	5.89	14.18	1.04	0.96	6.24	0.78
P12_post	5454394	2336	45.21	3.57	13.39	1.04	1	4.42	0.57
P14_post	5017370	2908	44.91	5.89	14.18	1.04	0.96	6.24	0.78
P14_pre	5133738	2774	43.55	4.58	12.46	1.03	0.96	6.13	0.77
P15_post	3554068	5912	43.45	4.44	12.22	1.04	0.96	6.77	0.78
P15_pre	5780539	14423	43.81	4.39	12.68	1.05	0.92	8.33	0.87
P16_post	2901936	194	42.87	4.09	11.58	1.03	0.94	3.9	0.74
P16_pre	6553910	3707	42.75	3.72	10.6	1.04	0.99	5.4	0.66
P18_post	2860836	1684	42.82	4.39	11.85	1.04	0.91	6.45	0.87
P18_pre	3899503	10160	43.09	4.2	11.89	1.05	0.88	8.35	0.9
P19_post	2878119	14518	42.55	3.99	12.03	1.04	0.92	8.28	0.86
P19_pre	3749427	4746	42.65	4	12.11	1.04	0.94	6.86	0.81
P20_post	2531512	3563	43.14	4.56	12.25	1.04	0.91	7.16	0.88
P21_pre	2335313	4977	43.16	4.55	12.39	1.04	0.88	7.67	0.9

Supplementary Table 3. Predictive values of putative biomarkers.

	MPR (n = 5)		nMPR (n = 14)		P-value	PPR+		nPPR+		P-value
	PDL1≥1	PDL1<1	PDL1≥1	PDL1<1		PDL1≥1	PDL1<1	PDL1≥1	PDL1<1	
PD-L1	3	2	0	13	0.0103	3	5	0	10	0.0686
	MSI-H	MSS	MSI-H	MSS		MSI-H	MSS	MSI-H	MSS	
MSI	3	2	0	14	0.0088	3	5	0	11	0.0578
	TMB-H	TMB-L	TMB-H	TMB-L		TMB-H	TMB-L	TMB-H	TMB-L	
TMB	4	1	1	13	0.0049	5	3	0	11	0.0048
	TNB-H	TNB-L	TNB-H	TNB-L		TNB-H	TNB-L	TNB-H	TNB-L	
TNB	3	2	0	14	0.0088	3	5	0	11	0.0578
RREB1	RREB1-mt	RREB1-wt	RREB1-mt	RREB1-wt		RREB1-mt	RREB1-wt	RREB1-mt	RREB1-wt	
mutation	4	1	0	14	0.001	4	4	0	11	0.0181
SSPO	SSPO-mt	SSPO-wt	SSPO-mt	SSPO-wt		SSPO-mt	SSPO-wt	SSPO-mt	SSPO-wt	
mutation	4	1	1	13	0.0049	4	4	1	10	0.1108
PD-L1 + RREB1	Positive	Negative	Positive	Negative		Positive	Negative	Positive	Negative	
mutation	5	0	0	14	<0.0001	5	3	0	10	0.0065

TMB-H is defined as TMB > 10; TNB-H is defined as TNB > 7; P-values are caculated by Fisher's exact tests.
