# Supplementary material for: Neoadjuvant therapy with immune checkpoint blockade, antiangiogenesis, and chemotherapy for locally advanced gastric cancer
Source: Nat Commun. 2023 Jan 3;14:8. doi: 10.1038/s41467-022-35431-x (PMC9810618; doi:10.1038/s41467-022-35431-x)
Supplement: Supplementary file 3 — Description of Additional Supplementary Files [file 41467_2022_35431_MOESM3_ESM.pdf]

Title: Supplementary Data 1:
Description: Somatic mutations.

Title: Supplementary Data 2:
Description: Gene mutation frequencies in MPR and non-MPR groups.

Title: Supplementary Data 3:
Description: Quality control of Whole-exome sequencing data.
