# Supplementary material for: Neoadjuvant therapy with immune checkpoint blockade, antiangiogenesis, and chemotherapy for locally advanced gastric cancer
Source: Nat Commun. 2023 Jan 3;14:8. doi: 10.1038/s41467-022-35431-x (PMC9810618; doi:10.1038/s41467-022-35431-x)
Supplement: Supplementary file 7 — Reporting Summary [file 41467_2022_35431_MOESM7_ESM.pdf]

nature portfolio

Corresponding author(s): Lian Liu

Last updated by author(s): Nov 4, 2022

Reporting Summary

Nature Portfolio wishes to improve the reproducibility of the work that we publish. This form provides structure for consistency and transparency in reporting. For further information on Nature Portfolio policies, see our Editorial Policies and the Editorial Policy Checklist.

Statistics

For all statistical analyses, confirm that the following items are present in the figure legend, table legend, main text, or Methods section.

n/a	Confirmed	
☐	☒	The exact sample size (n) for each experimental group/condition, given as a discrete number and unit of measurement
☐	☒	A statement on whether measurements were taken from distinct samples or whether the same sample was measured repeatedly
☐	☒	The statistical test(s) used AND whether they are one- or two-sided<br>*Only common tests should be described solely by name; describe more complex techniques in the Methods section.*
☐	☒	A description of all covariates tested
☐	☒	A description of any assumptions or corrections, such as tests of normality and adjustment for multiple comparisons
☐	☒	A full description of the statistical parameters including central tendency (e.g. means) or other basic estimates (e.g. regression coefficient) AND variation (e.g. standard deviation) or associated estimates of uncertainty (e.g. confidence intervals)
☐	☒	For null hypothesis testing, the test statistic (e.g. F, t, r) with confidence intervals, effect sizes, degrees of freedom and P value noted<br>*Give P values as exact values whenever suitable.*
☒	☐	For Bayesian analysis, information on the choice of priors and Markov chain Monte Carlo settings
☒	☐	For hierarchical and complex designs, identification of the appropriate level for tests and full reporting of outcomes
☒	☐	Estimates of effect sizes (e.g. Cohen's d, Pearson's r), indicating how they were calculated

Our web collection on statistics for biologists contains articles on many of the points above.

Software and code

Policy information about availability of computer code

Data collection	Data was collected with:<br>Microsoft Excel version 2019<br>NextSeq 550AR platform<br>PerkinElmer Vectra 3.0
Data analysis	Data were analyzed with:<br>Graph Pad Prism (v. 9.0)<br>SOAPnuke (v1.5.6)<br>Sambamba (v0.5.4)<br>BWA (v0.7.12)<br>Varscan (v2.4)<br>MuTect2 (v4.1.8.1)<br>Strelka (v2.9.10)<br>snpEff (v4.3)<br>POLYSOLVER (v1.0)<br>Bwakit (v0.7.11)<br>MSIsensor (v0.6)<br>PyClone (v0.13.1)<br>Kallisto (v0.46.2)<br>UCSC Xena Browser (https://xenabrowser.net/)<br>KOBAS-i webtool (http://bioinfo.org/kobas)<br>MiXCR (v2.1.10)<br>VDJtools (v1.2.1)

R (v3.6.1)
dNdScv (v0.1.0)

For manuscripts utilizing custom algorithms or software that are central to the research but not yet described in published literature, software must be made available to editors and reviewers. We strongly encourage code deposition in a community repository (e.g. GitHub). See the Nature Portfolio guidelines for submitting code & software for further information.

Data

Policy information about availability of data

All manuscripts must include a data availability statement. This statement should provide the following information, where applicable:

- Accession codes, unique identifiers, or web links for publicly available datasets
- A description of any restrictions on data availability
- For clinical datasets or third party data, please ensure that the statement adheres to our policy

WES, transcriptome sequencing, and TCR sequencing data generated in this study have been deposited in the Genome Sequence Archive under the accession code HRA002181 [https://ngdc.cncb.ac.cn/gsa/]. Access can be requested through the National Genomics Data Center [https://ngdc.cncb.ac.cn/] as detailed in the instructions. Access is granted for the entire period specified in the users' applications. Mutation data of the TCGA Stomach Cancer and TCGA Pan-Cancer cohorts were from the UCSC Xena [https://xena.ucsc.edu/]. The reference datasets included GENCODE (v38) database [https://www.gencodegenes.org/human/], dbSNP (version 147) [https://www.ncbi.nlm.nih.gov/snp], 1000G (phase3_release_v5) [https://www.internationalgenome.org], CLINVAR (version 151) [https://www.ncbi.nlm.nih.gov/clinvar], COSMIC (version 81) [https://cancer.sanger.ac.uk], and IMGT [https://www.imgt.org/]. CT scan and pathological imaging are not shared. The other individual de-identified participant data, Study Protocol, and Statistical Analysis Plan are available within 3 years after this paper's publication. Qualified researchers may request access to individual patient-level clinical data by contacting lianliu@sdu.edu.cn. The remaining data are available within the Article, Supplementary Information, or Source Data file. Source data are provided with this paper.

Field-specific reporting

Please select the one below that is the best fit for your research. If you are not sure, read the appropriate sections before making your selection.

☒ Life sciences ☐ Behavioural & social sciences ☐ Ecological, evolutionary & environmental sciences

For a reference copy of the document with all sections, see nature.com/documents/nr-reporting-summary-flat.pdf

Life sciences study design

All studies must disclose on these points even when the disclosure is negative.

Sample size	Since there were no previous reports on the efficacies of ICI or ICI-based neoadjuvant therapy for gastric cancer, formal sample size calculation cannot be performed. Based on the expected inclusion of patients in our single medical center in 2-3 years, the study aimed to include 30 patients.
Data exclusions	In total, 25 patients were screened for eligibility, and all were included and treated with neoadjuvant therapy. During the treatment, 2 refused surgery, and 3 were re-graded into stage IV due to peritoneal metastasis found during surgery.
Replication	Due to clinical sample availability, all lab examinations, including whole-exome sequencing, transcriptome sequencing, immunohistochemistry, and multiplex immunofluorescence, were performed once.
Randomization	This is a prospective, open-labeled, single-arm phase 2 trial.
Blinding	This is a prospective, open-labeled, single-arm phase 2 trial.

Reporting for specific materials, systems and methods

We require information from authors about some types of materials, experimental systems and methods used in many studies. Here, indicate whether each material, system or method listed is relevant to your study. If you are not sure if a list item applies to your research, read the appropriate section before selecting a response.

Materials & experimental systems

n/a	Involved in the study	
☐	☒	Antibodies
☒	☐	Eukaryotic cell lines
☒	☐	Palaeontology and archaeology
☒	☐	Animals and other organisms
☐	☒	Human research participants
☐	☒	Clinical data
☒	☐	Dual use research of concern

Methods

n/a	Involved in the study	
☒	☐	ChIP-seq
☒	☐	Flow cytometry
☒	☐	MRI-based neuroimaging

Antibodies

Antibodies used	Antibodies used for IHC:<br>anti-PD-L1 clone: 22C3; Provider: Dako; delusion: already diluted.<br>Anti-HER2 clone: 4B5; Provider: Ventana; dilusion: already diluted.<br><br>Antibodies used for multiplex immunofluorescence:<br>Anti-PD-1 clone: UMAB199; Provider: Zsbio; Lot#: ZM-0381; delusion: already diluted.<br>Anti-PD-L1 clone: E1L3N; Provider: Cell Signaling Technology; Lot #: 13684; dilusion: 1:200.<br>Anti-CD8 clone: C8/468 + C8/144B; Provider: Abcam; Lot#: ab199016; dilution: 1:200.<br>Anti-CD68 clone: KP1; Provider: Abcam; Lot#: ab955; dilution: 1:100.<br>Anti-FoxP3 clone: 236A/E7; Provider: Abcam; Lot#: ab20034; dilution: 1:100.<br>Anti-CK clone: AE1/AE3; Provider: Zsbio; Lot#: ZM-0069; dilusion: 1:200.
Validation	Specificity for each staining has been validated previously.<br><br>Validation of the use of C8/468 + C8/144B has been provided by the manufacture's website: https://www.abcam.com/cd8-alpha-antibody-c8468--c8144b-ab199016.html.<br>Validation of the use of KP1 has been provided by the manufacture's website: https://www.abcam.com/CD68-antibody-KP1-ab955.html.<br>Validation of the use of UMAB199 has been provided by the manufacture's website: http://www.zsbio.com/product/ZM-0381.<br>Validation of the use of AE1/AE3 has been provided by the manufacture's website: http://www.zsbio.com/product/ZM-0069.<br>Validation of the use of E1L3N has been provided by the manufacture's website: https://www.cellsignal.com/products/primary-antibodies/pd-l1-e1l3n-xp-rabbit-mab/13684.<br>Validation of the use of 236A/E7 has been provided by the manufacture's website: https://www.abcam.com/foxp3-antibody-236ae7-ab20034.html.

Human research participants

Policy information about studies involving human research participants

Population characteristics	The patients were between 48 and 70-year old, 19 male and 6 female. All were locally advanced gastric cancer by histology, 11 cT4aN+ and 14 cT4bN+, and Eastern Cooperative Oncology Group (ECOG) score 0-1. They did not have distant metastasis by physical examination and CT scan, chemotherapy/radiotherapy/immunotherapy history, or active autoimmune disease.
Recruitment	Patients were all recruited from our center by doctors in the Department of General Surgery or Medical Oncology at Shandong University Qilu Hospital, China. Patients were recruited according to predefined inclusion and exclusion criteria in the study protocol. All patients are cT4 (cT4a or cT4b) and N+, which may lead to inferior outcomes compared to the other neoadjuvant trials with locally advanced gastric cancer (generally cT3-4 or cN+). In addition, the small sample size and single-arm design may also introduce inclusion bias.
Ethics oversight	The study was conducted according to the Declaration of Helsinki and Good Clinical Practice and monitored by the Medical Ethical Committee of Shandong University Qilu Hospital. This protocol was approved by the Medical Ethical Committee of Shandong University Qilu Hospital (Number: 2018214). This clinical trial was registered at https://www.clinicaltrials.gov/ before patient enrollment (clinical trial identifier NCT03878472). A total of 25 patients were enrolled, and all patients provided written informed consent.

Note that full information on the approval of the study protocol must also be provided in the manuscript.

Clinical data

Policy information about clinical studies

All manuscripts should comply with the ICMJE guidelines for publication of clinical research and a completed CONSORT checklist must be included with all submissions.

Clinical trial registration	This clinical trial was registered at https://www.clinicaltrials.gov/ before patient enrollment (clinical trial identifier NCT03878472).
Study protocol	The study protocol is uploaded with this submission and made available upon request.
Data collection	The first patient was enrolled on May 18, 2019, and the last was enrolled on August 25, 2020. Data collection for the current analysis was performed on July 31, 2022. Clinical data were collected from the time of informed consent and up to 3 years after the first dose of treatment. Toxicity evaluation starts from the first course of treatment to 30 days after the last treatment. Blood samples were collected at the Department of Medical Oncology or Gastrointestinal Surgery, Shandong University Qilu Hospital, China. Tissue samples were collected from the Department of Pathology, Shandong University Qilu Hospital, China.
Outcomes	Primary endpoints include pathological responses and their potential biomarkers. Pathological responses are evaluated by pathologists according to the proportions of residual viable tumor cells in tumor beds. Biomarkers are identified from immunohistochemistry, whole-exome sequencing, transcriptome sequencing, and TCR sequencing.<br>Secondary endpoints include safety, objective response, 1-year PFS rate, and 1-year OS rate. Toxicity is monitored closely with changes of physical examination, clinical laboratory analyses, and reported adverse events up to 30 days after the last treatment, according to CTCAE 5.0. Objective responses are evaluated by oncologists and radiologists according to RECIST 1.1. Disease-free survival and overall survival are also followed up.
